# Therapeutic Advances in Targeting the Amyloid-β Pathway for Alzheimer’s Disease

**DOI:** 10.3390/brainsci15101101

**Published:** 2025-10-13

**Authors:** Beiyu Zhang, Yunan Li, Huan Li, Xinai Shen, Zheying Zhu

**Affiliations:** Division of Molecular Therapeutics and Formulations, School of Pharmacy, The University of Nottingham, University Park Campus, Nottingham NG7 2RD, UK; beiyu.zhang@nottingham.ac.uk (B.Z.); alyhl28@nottingham.ac.uk (H.L.); xinai.shen@nottingham.ac.uk (X.S.)

**Keywords:** Alzheimer’s disease, amyloid-β hypothesis, amyloid precursor protein (APP) processing, biomarkers, small-molecule drugs, β-secretase 1 (BACE1) inhibitors, γ-secretase modulators, monoclonal antibodies, anti–amyloid-β (Aβ) vaccines, gene therapy

## Abstract

Alzheimer’s disease (AD) is the most common cause of dementia, characterized by progressive cognitive decline and neuropathological hallmarks, including amyloid-β (Aβ) plaques, neurofibrillary tangles (NFTs), and neurodegeneration. Since the amyloid cascade hypothesis was proposed, Aβ has remained a central therapeutic target, with interventions aiming to reduce Aβ production, aggregation, or downstream toxicity. This review first outlines the historical development of the Aβ hypothesis and the two major APP processing pathways (α-cleavage and β-cleavage), highlighting the role of biomarkers in early diagnosis, patient stratification, and regulatory approval. We then summarize the development and clinical outcomes of anti-Aβ small-molecule drugs, including β-secretase inhibitors, γ-secretase modulators, Aβ aggregation inhibitors, receptor/synapse modulators, and metabolic or antioxidant modalities. We further review the progression of biologic therapies, with a particular focus on monoclonal antibodies, vaccines, and emerging gene-silencing strategies, such as small interfering RNA (siRNA) and antisense oligonucleotides. Finally, we discuss future perspectives, including next-generation biologics, multi-target approaches, optimized delivery platforms, and early-prevention strategies. Collectively, these efforts underscore both the challenges and opportunities in translating anti-Aβ therapies into meaningful clinical benefits for patients with AD.

## 1. Introduction of AD

Alzheimer’s disease (AD) is a degenerative condition of the nervous system characterized by a progressive decline in memory and cognitive function, followed by behavioral changes and the loss of functional abilities. The impact of AD is recognized by the World Health Organization, which acknowledges it as a growing global health concern affecting individuals, caregivers, and society at large. Recent projections suggest that by 2050, dementia prevalence will increase twofold in Europe and threefold globally [1]. When AD is defined using pathological criteria rather than clinical diagnosis, the projected prevalence is approximately three times higher [1]. Currently approved small-molecule drugs—such as acetylcholinesterase inhibitors and NMDA receptor antagonists—primarily provide symptomatic relief. In contrast, newer investigational Aβ-targeting agents, including β-secretase inhibitors, γ-secretase modulators, and monoclonal antibodies against aggregated Aβ, aim to modify the disease process at upstream pathological stages [2]. Aggregated β-amyloid (Aβ), rather than monomeric Aβ, is a major histopathological hallmark of AD [3,4]. It is associated with age-related cognitive decline, neurotoxicity, and the formation of neurofibrillary tangles (NFT) [5,6]. Deposition of cerebral amyloid-β (Aβ) fibrils may begin decades before clinical symptoms appear [7]. In 1991, three independent groups identified Aβ as a central factor in AD pathogenesis [8,9,10]. In familial AD, autosomal-dominant mutations in genes encoding *amyloid precursor proteins*—*APP*, *presenilin-1* (*PSEN1*), and *presenilin-2* (*PSEN2*)—were shown to drive abnormal Aβ production [10,11,12]. Similarly, individuals with Down syndrome, who carry an additional copy of the APP gene, produce excess amyloid and are at higher risk of AD in midlife [13]. Collectively, these genetic and pathological findings form the basis of the amyloid hypothesis, which remains the dominant framework for explaining AD etiology and has guided the development of current therapeutic strategies [14]. However, recent analyses, such as the Am J Pathol. (2024) review, have further highlighted both the long-standing mechanistic evidence supporting Aβ as a pathogenic driver, while also emphasizing that clinical outcomes from Aβ-targeted interventions remain modest, suggesting that amyloid pathology may represent only one component of a more complex disease process [15].

Decades of drug development targeting Aβ yielded repeated trial failures, casting doubt on the amyloid hypothesis [15]. However, the past five years have seen a turning point. Encouraging trial results and regulatory approvals of several Aβ-targeting biologics represent a milestone in AD therapy. These agents, which can clear brain amyloid, are the first to show disease-modifying potential, renewing optimism in Aβ as a viable therapeutic target. This represents a milestone in AD drug development, as no other pathological pathway has yet achieved regulatory validation of clinical efficacy [14]. Aducanumab (Aduhelm) was granted accelerated approval in 2021, based on its ability to reduce Aβ plaque burden as a surrogate endpoint [16]. Lecanemab (Leqembi) initially received accelerated [17] approval in early 2023 [18] and was subsequently converted to traditional approval in July 2023, supported by the CLARITY-AD trial demonstrating verifiable clinical benefit. Most recently, donanemab (Kisunla) was approved in 2024 for the treatment of early AD, marking the third antibody to reach full regulatory endorsement [19].

The significance of these approvals is twofold. First, they collectively establish Aβ as the only therapeutic target to date that has completed the full regulatory “loop” from biomarker engagement to confirmed clinical efficacy. Second, they underscore why the current AD therapeutic landscape is centered on Aβ, despite decades of controversy and the failures of numerous other approaches [20]. So we focus on Aβ—tracing the trajectory of the amyloid hypothesis from its molecular origins to its clinical validation, analyzing both the successes and setbacks, and exploring the next wave of Aβ-targeting strategies, particularly biologics and nucleic acid therapeutics.

### 1.1. The Aβ Hypothesis

#### 1.1.1. The Development of the Aβ Hypothesis [21]

**Figure 1:**
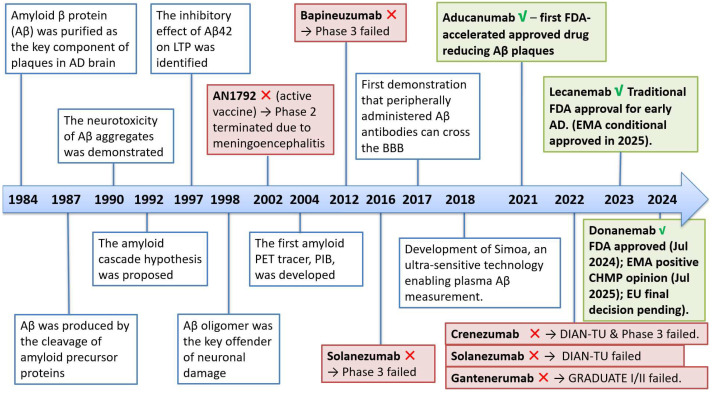
Timeline of Aβ hypothesis development and therapeutic milestones in AD. Scientific discoveries (blue boxes) established the amyloid cascade hypothesis and advanced diagnostic tools. Multiple Aβ-targeted immunotherapies (red boxes) failed in clinical trials, including AN1792, bapineuzumab, solanezumab, crenezumab, and gantenerumab. Recent breakthroughs (green boxes) include the FDA approvals of aducanumab (2021), lecanemab (2023), and donanemab (2024), marking a new era for Aβ-directed disease-modifying therapies.

Aβ accumulation has long been viewed as a key driver of AD pathology (the “amyloid cascade hypothesis”). In 1984, Glenner and Wong identified amyloid β (Aβ) as the principal constituent of the extracellular amyloid plaques characteristic of AD [22]. And in 1992, Hardy and Higgins formulated the “amyloid cascade hypothesis”, proposing that abnormal Aβ accumulation initiates a cascade leading to tau hyperphosphorylation, synaptic dysfunction, neuronal death, and ultimately dementia [23]. Over time, extensive experimental and genetic evidence refined the Aβ hypothesis (key milestones summarized in Figure 1) [24]. Early studies demonstrated the neurotoxicity of Aβ aggregates [22] and clarified that Aβ is generated by cleavage of the amyloid precursor protein (APP) [25]. The first amyloid PET tracer, PIB, was developed in 2004 [26], followed by ultrasensitive plasma biomarker assays, such as Simoa in 2018, enabling more precise staging and monitoring [27]. Early immunotherapy attempts faced safety and efficacy limitations [28,29,30,31,32,33]. More recently, second-generation antibodies have shown clinical impact (to discuss in Section 4).

This historical foundation has been further reinforced by the NIA–AA ATN research model, which redefines AD as a biological construct, classifying individuals according to biomarkers of Aβ deposition (A), pathologic tau protein (T), and neurodegeneration (N). This tripartite system, assessed via fluid biomarkers, PET imaging, and neuroimaging, enables objective staging across the disease continuum, facilitates earlier and more precise diagnosis, harmonizes research cohorts, and supports the development of targeted interventions aligned with underlying pathology [34]. Therapeutically, several strategies have been explored to lower Aβ, including inhibition of β- and γ-secretases to block peptide generation, prevention of Aβ aggregation, and both active and passive immunization [35,36,37,38]. Both passive and active immunization of transgenic mice against β-amyloid can reverse neuropathology and improve pathologic learning and memory behaviors [39,40,41,42,43]. In a human patient with AD, immunization against β-amyloid was associated with sizable brain areas devoid of β-amyloid, reduced neuritic pathology, reduced astrocytosis, and microglial cells filled with β-amyloid [44], as predicted by previous immunization experiments in transgenic mice. These insights directly informed the development of monoclonal antibody therapies, culminating in the recent, and widely debated, FDA accelerated approvals of aducanumab and lecanemab, based primarily on their ability to reduce amyloid plaque burden [45].

#### 1.1.2. APP Processing: Amyloidogenic and Non-amyloidogenic Pathways

The accumulation of Aβ peptide as plaques in the brain is a prominent pathological hallmark of AD [46]. Aβ is generated from the APP [47], which is a member of a conserved protein family comprising APP-like protein 1 (APLP1) and APLP2 [48]. APP is a transmembrane protein widely distributed throughout the body, characterized by a single-pass structure encompassing an extracellular domain, a hydrophobic transmembrane domain, and an intracellular domain. The proteolytic cleavage of APP can be facilitated by various secretase complexes, operating through two distinct pathways [49]. One is a non-amyloidogenic pathway that will not generate Aβ using α- and γ-secretase and the other is amyloidogenic pathway that generates the Aβ peptide using β- and γ-secretase. Typically, the majority of APP follows the non-amyloidogenic pathway, where the APP is cleaved at the α-secretase site located at amino acid 17 of the Aβ domain consisting of 40–42 amino acids. This cleavage process yields two fragments: sAPPα and a CTF-α [50]. The cleavage of APP by α-secretase prevents the production of Aβ due to its cleavage site being located within the Aβ domain, specifically at the Lys16-Leu17 bond. This process results in the release of a soluble ectodomain of APP known as sAPPα [51]. sAPPα has been discovered to possess a significantly higher neuroprotective ability against excitotoxicity and Aβ toxicity compared to the slightly larger sAPPβ, which is specific to β-secretase. The potency of sAPPα in safeguarding neurons is reported to be 100 times greater [52]. α-secretase is a zinc metalloproteinase, and α-secretase-like activity has been observed in various members of the ADAM family, with ADAM9, ADAM10, and ADAM17 being proposed as α-secretase [53].

**Figure 2:**
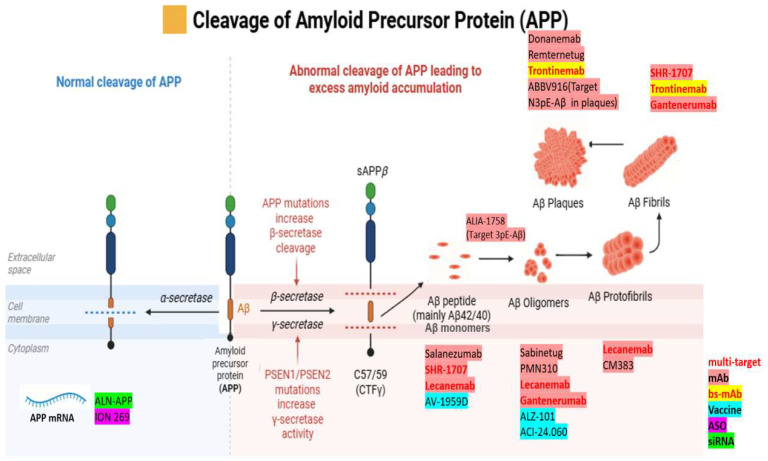
Schematic representation of APP processing and therapeutic interventions targeting different Aβ species. (Created in https://BioRender.com). The left panel illustrates the normal α-secretase cleavage pathway, while the right panel shows abnormal β- and γ-secretase cleavage leading to excess amyloid accumulation and aggregation into monomers, oligomers, protofibrils, fibrils, and plaques. Drugs are positioned according to their primary target species as follows: monoclonal antibodies (mAb, red), bispecific monoclonal antibodies (bs-mAb, yellow), vaccines (cyan), antisense oligonucleotides (ASO, magenta), and siRNA (green). Orange-red labels indicate multi-target agents acting on more than one Aβ species.

Aducanumab (Figure 2) binds Aβ fibrils and plaques (epitope amino acids 3–7 of Aβ) [54]; Lecanemab (Figure 2) binds soluble protofibrils and aggregates (epitopes 1–16 and 21–29) [55]; Donanemab (Figure 2) targets N-terminal pyroglutamate-modified Aβ present in plaques [56]. These antibodies facilitate clearance of Aβ via microglial phagocytosis or other mechanisms, aiming to slow AD progression [57].

In Figure 2, β-processing is the first step of APP amyloidogenic pathway using β-secretase, which generates Aβ. β-secretase is a membrane-bound aspartyl protease protein that can first cleave the precursor protein at the beginning of the Aβ domain resulting in a C-terminal fragment of CTF-β and sAPPβ [58]. CTF-β is further cleaved by γ-secretases and generates extracellular Aβ peptide fragments and APP intracellular domain (AICD) [59]. β-APP-Cleaving Enzyme (BACE1) is a major β-secretase involved in APP metabolism. Elevated levels of BACE1 protein and activity have been observed in the brain regions affected by AD in several studies [60,61]. Many studies show that BACE1 is an efficient therapeutic target. There are several small molecules as BACE1 inhibitors, among them, four drug candidates are currently in clinical trials [58]. BACE1 inhibitor is able to prevent the formation of Aβ at early APP processing and improves the neurological function. Furthermore, in AD mice models, the absence of BACE1 has been demonstrated to alleviate cholinergic dysfunction, neuronal loss, and memory impairments, accompanied by a significant reduction in Aβ40/42 levels [62]. Collectively, these findings support BACE1 as a promising target for the therapy of AD. Following the cleavage processes of α- and β-cleavage, APP generates CTFs, which are referred to as CTF-α and CTF-β, and undergo subsequent cleavage by γ-secretase [48].

In contrast, α-secretase cleaves APP within the Aβ domain, initiating the non-amyloidogenic pathway and producing sAPPα and CTF-α (C83). Subsequent γ-secretase cleavage of CTF-α releases a short p3 peptide, which is rapidly degraded and is generally considered non-toxic, together with AICD [63]. Various biochemical studies have provided evidence indicating that γ-secretase function is attributed to a high molecular weight assembly comprising a minimum of four constituents: presenilin (PSEN, PSEN1 or PSEN2), Nicastrin, anterior pharynx-defective-1 (APH-1), and presenilin enhancer-2 (PEN-2) [64]. Presenilin, which forms a considerably stable protein complex with substantial relative molecular mass plays a critical role in facilitating the activity of γ-secretase [65]. This intricate assemblage collaborates with a set of auxiliary proteins [63]. Familial Alzheimer’s disease (FAD) is associated with mutations in genes encoding γ-secretase, specifically presenilin1 and presenilin 2. These mutations enhance amyloidogenic APP processing, ultimately leading to excessive accumulation of Aβ peptides and amyloid plaque formation. In cases of dominantly inherited AD, various mutations in presenilin (PSEN) have been observed, causing a shift in the primary cleavage site of γ-secretase from position 40 to 42. As a result, longer Aβ peptides are generated, which have a greater tendency to rapidly aggregate and become the predominant constituents of amyloid plaques [65].

#### 1.1.3. Biomarkers in Clinical Trials and Therapy

Biomarkers are now central to AD drug development. They not only enable early and accurate diagnosis but also ensure that anti-Aβ therapies are tested in patients with confirmed amyloid pathology. In clinical trials, biomarkers serve multiple functions as follows: as inclusion criteria for patient selection (e.g., requiring a minimum amyloid burden), as pharmacodynamic readouts for dose optimization (quantifying plaque removal at a given dose), and as tools for safety monitoring (e.g., MRI for ARIA, CSF assays for inflammatory markers). Importantly, amyloid PET reduction has been accepted as a surrogate endpoint in regulatory pathways, supporting accelerated approval of anti-Aβ monoclonal antibodies. Collectively, these advances in biomarker science complement therapeutic progress and are pushing the field toward earlier intervention, with the ultimate goal of treating AD at a preclinical stage before irreversible neurodegeneration occurs [66].

## 2. Small-Molecule Therapeutics in AD: Symptomatic Treatments and Aβ-Targeting Agents

### 2.1. Agents for Symptomatic Treatment

These medications are generally divided into two categories: cholinesterase inhibitors and NMDA receptor antagonists. However, except for the symptomatic cholinesterase inhibitors (donepezil [67], rivastigmine [68], galantamine [69]) and the NMDA receptor antagonist memantine [70], no small molecule has demonstrated a disease-modifying effect [71]. But these pharmacological treatments for AD primarily target the symptoms of the disease rather than its root causes [2]. In contrast, more recent investigational agents have focused on disease modification through targeting amyloid β (Aβ), including β-secretase inhibitors and γ-secretase modulators.

### 2.2. β-Secretase (BACE1) Inhibitors

Given that Aβ is produced by sequential cleavage of APP by β- and γ-secretases, these enzymes became attractive therapeutic targets. However, despite robust preclinical efficacy, clinical trials of secretase inhibitors have repeatedly failed. In particular, β-secretase (BACE1), which is the most extensively investigated small-molecule strategy, has consistently failed in clinical development for AD. Although BACE1 inhibitors effectively lowered cerebrospinal fluid (CSF) and plasma Aβ levels, none demonstrated clinical benefit, and many were associated with unacceptable toxicities [72]. Verubecestat (MK-8931, Merck, Rahway, NJ, USA), the first small-molecular BACE1 inhibitor with oral availability and blood–brain barrier permeability [73], which advanced to Phase III trials in both mild-to-moderate AD (EPOCH) and prodromal AD (APECS), showed no efficacy and even cognitive worsening, with adverse events, including weight loss, psychiatric symptoms, and dermatological changes [74]. Lanabecestat (AZD3293/LY3314814, AstraZeneca, Cambridge, UK/Eli Lilly, Indianapolis, IN, USA) was discontinued in 2018 after futility analyses of two large Phase III trials (AMARANTH and DAYBREAK-ALZ) confirmed lack of benefit [75]. Atabecestat (JNJ-54861911, Janssen, New Brunswick, NJ, USA) was terminated in 2018 because of hepatotoxicity [76], and umibecestat (CNP520, Novartis, Basel, Switzerland/Amgen, Thousand Oaks, CA, USA) was halted in 2019 after showing cognitive decline and weight loss in cognitively unimpaired APOE ε4 carriers [21]. Similarly, elubecestat (E2609, Eisai, Tokyo, Japan) was abandoned after Phase III futility analyses [77]. Collectively, these failures underscore the challenge of long-term BACE1 inhibition, as the enzyme has diverse physiological substrates beyond APP, including roles in myelination and synaptic function, leading to mechanism-based toxicities [72,74]. Overall, BACE1 inhibitor programs have failed primarily due to toxicity and lack of clinical benefit. Therefore, although small molecules have contributed to symptomatic management in AD, none of the BACE1-targeting approaches have yet demonstrated disease-modifying efficacy.

### 2.3. γ-Secretase Inhibitors/Modulators

γ-secretase inhibitors (GSIs) have long been investigated as potential therapeutic approaches for AD due to their ability to inhibit Aβ production [66]. Semagacestat (LY450139), one of the first GSIs to enter late-stage trials, reduced plasma and CSF Aβ levels in early studies. However, two Phase 3 trials conducted in mild-to-moderate AD (2008–2010) were halted after showing no clinical benefit and, instead, accelerated cognitive decline, along with significant adverse events, such as skin cancers and gastrointestinal symptoms [78]. These failures highlighted the limitations of broad γ-secretase inhibition, particularly the off-target effects arising from interference with Notch signaling. In an effort to improve selectivity, Avagacestat (BMS-708163) [79], an arylsulfonamide GSI with greater specificity for APP over Notch, was developed. Although it successfully reduced CSF Aβ in animal models without Notch-related toxicity and was initially considered promising, its Phase 2 trials (2010–2012) were terminated due to gastrointestinal and dermatological adverse effects [79]. Together, these outcomes illustrate that, despite improved selectivity, GSIs as a class have faced insurmountable safety and efficacy challenges in AD therapy. However, more recently, γ-secretase modulators (GSMs), such as RO7269162, have emerged as an alternative strategy that shifts cleavage preference rather than fully inhibits enzymatic activity [80]. RO7269162 is considered one of the most advanced GSM candidates in development, and will be discussed in more detail in Section 3.3.

Although β- and γ-secretase inhibitors have largely failed due to insufficient selectivity, off-target toxicities, and lack of clinical benefit, small-molecule strategies have not been abandoned altogether. Instead, the focus has shifted from “blocking upstream production” to “modulating downstream aggregation, synaptic signaling, and cellular resilience.” Recent pipeline agents illustrate this trend: compounds targeting Aβ oligomerization, Aβ–receptor interactions, or mitochondrial metabolism are being clinically tested, often in genetically defined subgroups, such as APOE4/4 carriers. These approaches aim to retain the advantages of oral administration and broad accessibility, while avoiding the mechanistic pitfalls of secretase inhibition.

Nevertheless, efforts to develop small molecules have not ceased; instead, the focus has shifted from non-selective blockade of APP processing toward agents that stabilize Aβ, modulate its receptor interactions, or enhance cellular resilience.

## 3. Small-Molecular Drugs in Human Clinical Trials

### 3.1. Aβ Aggregation Modulators

ALZ-801 (valiltramiprosate) is an oral, brain-penetrant prodrug of tramiprosate that inhibits Aβ42 oligomer formation by stabilizing soluble monomers. Unlike antibody therapies, it can be administered orally (265 mg twice daily) and is being developed as a precision medicine for APOE4 carriers [81]. In a two-year Phase 2 study in early AD patients carrying APOE4 alleles, ALZ-801 significantly reduced plasma p-tau181 (−31%), slowed hippocampal atrophy, and stabilized memory performance, without evidence of ARIA or vasogenic edema, supporting its disease-modifying potential [82]. A pivotal Phase 3 trial (APOLLOE4) is ongoing in 325 APOE ε4/ε4 homozygotes with early AD, powered to detect clinically meaningful benefits on cognition and function [81]. If successful, ALZ-801 may become the first safe and effective oral disease-modifying therapy for this high-risk genetic subgroup.

Contraloid acetate (PRI-002, also known as RD2) is an all-D-enantiomeric peptide developed by Priavoid GmbH to disassemble toxic Aβ oligomers into non-toxic monomers [83], thereby protecting synaptic function. In a randomized, double-blind Phase 1b trial (EudraCT 2020-003416-27; NCT04711486) conducted in 2020–2021, 20 patients aged 50–80 years with mild cognitive impairment (MCI) or mild AD received once-daily oral dosing of 300 mg PRI-002 for 28 days or placebo. The study met all primary safety and tolerability endpoints: the drug was well tolerated, no serious adverse events occurred, and importantly, no ARIA was observed on MRI. Pharmacokinetics were not influenced by age, sex, or body weight. Although no significant biomarker changes (p-tau, t-tau, Aβ species) were detected in CSF during this short trial, patients on PRI-002 showed improved performance in the CERAD word list test at Day 56 compared with placebo (*p* ≤ 0.05) [84]. These results suggest that PRI-002 is a safe, orally available peptide therapeutic with potential disease-modifying activity in early AD.

### 3.2. Aβ–Receptor/Synapse Modulators

ALX-001 (BMS-984923) is a first-in-class oral synapse-targeted mGluR5 silent allosteric modulator developed by Allyx Therapeutics. Unlike conventional anti-amyloid therapies that directly bind Aβ aggregates, ALX-001 blocks Aβ oligomer–mGluR5 synaptic receptor signaling, thereby protecting synaptic function and preventing downstream dysfunction and loss. This indirect approach aims to avoid the risks associated with direct amyloid binding, potentially offering improved efficacy and safety in an oral therapy. Phase 1 trials have demonstrated favorable safety and target engagement (confirmed by receptor occupancy imaging), and a Phase 2 program is now being prepared (NCT05804383). Early Phase 1b data presented at the AD/PD 2024 Conference (32 participants, single- and multiple-ascending dose cohorts) indicated that ALX-001 was well tolerated, with no clinically significant cognitive or psychiatric adverse effects and a 2.5-fold safety margin relative to the no-observed-adverse-effect level [85].

### 3.3. Metabolic/Antioxidant Modalities

MIB-626, a proprietary oral NMN-based NAD^+^ booster developed by Metro International Biotech, has been designed to enhance cellular NAD^+^ levels, potentially improving mitochondrial bioenergetics, mitigating oxidative stress, and supporting Aβ and tau protein clearance based on preclinical evidence of NAD^+^-mediated mitophagy and proteopathy mitigation. In a Phase 1 clinical trial, daily dosing resulted in a robust rise in circulating NAD^+^ levels (~3.7×) and excellent tolerability [86]. A Phase 2 trial is underway.

RQC (Resveratrol-Quercetin-Curcumin) is an orally administered antioxidant blend under investigation for its potential to prevent Aβ accumulation outside the brain, specifically in the retina [87], as a non-invasive proxy for Alzheimer’s pathology. Currently in a Phase I/II trial (NCT06470061), participants receive oral RQC to assess whether it can reduce retinal Aβ deposits, offering a novel avenue to monitor anti-amyloid effects. While preclinical studies support the neuroprotective and anti-amyloid potential of each constituent—resveratrol, quercetin, and curcumin—the clinical efficacy of RQC for AD remains to be established [88].

RO7269162 is an experimental small-molecule γ-secretase modulator (GSM) originally developed by F. Hofmann-La Roche AG, currently shown to be advancing through Phase 2 clinical development for AD. The compound is believed to act by modulating γ-secretase activity, thereby reducing the production of toxic β-amyloid peptides implicated in plaque formation. A Phase 1 trial (NCT06733298) assessed RO7269162’s pharmacokinetics, absorption, metabolism, and excretion in healthy males. The ongoing Phase 2a GABriella trial (NCT06402838), a randomized, double-blind, placebo-controlled, multiple-dose study, is evaluating the safety, tolerability, and biomarker effects, including changes in amyloid load (via PET imaging) and cerebrospinal fluid/plasma biomarkers, in participants either at risk for or in the prodromal stage of AD; this trial spans approximately 90 weeks with daily oral administration of RO7269162 or placebo, and it is expected to complete in October 2026 [89].

Table 1 summarizes representative small-molecule therapeutics under clinical evaluation for AD, including their mechanisms of action, routes of administration, clinical trial status, reported efficacy, and associated trial identifiers. Data are derived from ClinicalTrials.gov and published references up to 2025.

## 4. The Advances in Anti-Aβ Biologics Therapeutics

In contrast to small-molecule therapeutics, biologic agents have recently achieved landmark regulatory successes in AD. Monoclonal antibodies (mAbs) directed against Aβ plaques or protofibrils have been at the forefront of this progress. The first approval was aducanumab (Aduhelm), granted accelerated approval by the FDA in 2021 based on amyloid plaque reduction as a surrogate endpoint [91]. Monoclonal antibodies (mAbs) designed to bind Aβ and promote its clearance have been a major focus of AD therapeutics. Early-generation mAbs targeted soluble monomeric Aβ but failed to show clinical efficacy. Newer “second-generation” mAbs target aggregated forms of Aβ and have demonstrated the ability to remove plaques and modestly slow cognitive decline [20]. Lecanemab (Leqembi) received accelerated approval in January 2023 and full approval in July 2023 after the confirmatory CLARITY-AD trial showed slowed cognitive decline in early AD [92]. Donanemab (Kisunla) was approved in July 2024 for early symptomatic AD, supported by the TRAILBLAZER-ALZ 2 trial demonstrating significant benefit in slowing clinical decline [93]. Collectively, these approvals establish anti-Aβ mAbs as the first disease-modifying therapies for AD with regulatory validation of clinical benefit, setting the stage for further development of biologic modalities.

Table 2 summarizes ongoing and completed clinical trials of biologic drugs for AD, including monoclonal antibodies, active immunization vaccines, and nucleic acid–based biologics (siRNA/ASO). Information includes modality, disease stage, primary targets/epitopes, safety findings, trial identifiers, and trial/regulatory status.

### 4.1. Monoclonal Antibodies (mAbs)

Monoclonal antibodies (mAbs) designed to bind Aβ and promote its clearance have been a major focus of AD therapeutics. Early-generation mAbs targeted soluble monomeric Aβ but failed to show clinical efficacy. Newer “second-generation” mAbs target aggregated forms of Aβ and have demonstrated the ability to remove plaques and modestly slow cognitive decline [20].

First-generation mAbs, such as Solanezumab [114], bapineuzumab [29,115], crenezumab [116], aimed at Aβ monomers or generic epitopes did not significantly improve outcomes in Phase III trials despite lowering some biomarkers. For example, solanezumab (targeting soluble Aβ) showed no cognitive benefit in mild AD and failed to halt amyloid accumulation [114]. The failure of these antibodies suggested that targeting monomeric Aβ or treating later-stage patients was insufficient. Second-generation mAbs, such as Aducanumab, Lecanemab, Donanemab, Gantenerumab, were designed to target aggregated, pathogenic Aβ species (oligomers, fibrils, plaques) [117]. Anti-Aβ antibody therapy is frequently associated with amyloid-related imaging abnormalities (ARIA). ARIA is classified into two subtypes. ARIA-E (edema/effusion) reflects fluid leakage leading to vasogenic edema or sulcal effusion, whereas ARIA-H (hemosiderosis or microhemorrhages) denotes micro- or macrohemorrhages that appear as hypointense hemosiderin deposits on MRI. Among these, ARIA-E is the most commonly reported adverse event associated with anti-Aβ antibody therapy [118]. Despite these safety concerns, several monoclonal antibodies—such as aducanumab, lecanemab, and donanemab—have shown encouraging efficacy in recent clinical trials [92,96], each demonstrating distinct profiles in terms of amyloid clearance and ARIA incidence as follows:

Aducanumab is a human monoclonal antibody that selectively binds Aβ aggregates (soluble oligomers and insoluble fibrils) [119,120]. Once aducanumab binds Aβ plaques, microglia phagocytose them, potentially slowing AD progression [91]. Administered intravenously, this drug crosses the blood–brain barrier, binds to amyloid in the brain, and utilizes the immune system to remove amyloid proteins. It targets amyloid in patients with slowly progressing dementia [121]. In July 2015, an interim Phase 1b study showed plaque reduction and dose-dependent ARIA as the main safety concern [122]. Sevigny et al. published a phase Ib study in August 2016, based on one year of “monthly intravenous infusions” of aducanumab in patients with prodromal or mild AD, in which brain scans by molecular positron emission tomography (PET) imaging to measure amyloid plaque confirmed that one year of monthly IV aducanumab reduced Aβ in a dose- and time-dependent manner and slowed clinical decline, with ARIA as the main adverse event [91]. However, the main safety and tolerability findings are amyloid-related imaging abnormalities (ARIA) [91]. What is more, Biogen did not conduct the Phase II trials because the FDA did not require them, which led to criticism from some experts [123]. Based on these positive results, Biogen initiated two pivotal Phase III clinical trials: EMERGE and ENGAGE. These trials aimed to evaluate Aducanumab’s ability to preserve cognitive function in patients with early AD [124]. Despite mixed results from the EMERGE and ENGAGE trials, post hoc analyses suggested benefit in high-dose subgroups, supporting FDA Priority Review Thia contributed to the FDA’s acceptance of the BLA (Biologics License Application) [16]. The US Food and Drug Administration’s (FDA) accelerated approval of Biogen’s aducanumab in June 2021 has made it the first approved drug for slowing cognitive decline in AD patients and also the first new drug for this disease in nearly two decades [54]. However, due to limited uptake, payer restrictions, and continued controversy over its efficacy and safety, Biogen announced in January 2024 that it would discontinue marketing of Aduhelm (aducanumab) [125]. Aducanumab’s withdrawal illustrates the challenges of implementation, but its approval remains a milestone, demonstrating that disease modification in AD is achievable through Aβ immunotherapy.

Lecanemab (BAN2401): IgG1 mAb derived from the murine antibody mAb158 was designed to selectively target soluble Aβ protofibrils (large oligomers) with an epitope spanning Aβ residues 1–16 [126]. In a Phase 1 single- and multiple-ascending dose study (NCT01230853) involving 80 patients with mild-to-moderate AD, lecanemab showed good safety and tolerability across escalating doses, with rare SAEs and ARIA rates comparable to the placebo [127]. Study 201 (NCT01767311), a Phase 2 trial in 856 patients with MCI due to AD or mild AD confirmed by amyloid PET/CSF, assessed lecanemab’s safety, tolerability, and efficacy. At the highest dose (10 mg/kg biweekly), Bayesian analysis of ADCOMS at 12 months showed a 64% probability of >25% benefit versus placebo, below the 80% threshold. Nonetheless, secondary endpoints at 18 months indicated significant slowing of decline (30% in ADCOMS, 47% in ADAS-Cog14, 26% in CDR-SB), alongside reductions in brain Aβ and CSF p-tau. ARIA-E occurred in 9.9% of high-dose patients and in 14.3% of APOE ε4 carriers [128]. The Phase 3 Clarity AD trial (NCT03887455) enrolled 1906 patients with MCI due to AD or mild AD. Lecanemab reduced clinical decline on the primary endpoint, CDR-SB, by 27% at 18 months versus placebo. Secondary outcomes also favored lecanemab, with 26% slowing on ADAS-Cog14, 24% on ADCOMS, and 37% on ADCS-ADL-MCI. Brain amyloid burden decreased by 59.1 centiloids (77.9 → 18.8) from baseline. Safety analyses showed ARIA-E in 12.6% of patients (2.8% symptomatic) and ARIA-H in 17.3% (0.7% symptomatic). These findings supported FDA conversion of lecanemab to traditional approval [129]. It received FDA traditional approval in 2023 as the first *fully* approved amyloid-directed therapy after demonstrating clinical benefit [19]. Lecanemab’s success provided strong support for the amyloid hypothesis, though patients must be early in disease for efficacy.

Donanemab: IgG1 mAb recognizes the N-terminal pyroglutamate-modified Aβ (Aβ_p3–42) found in plaque cores [66]. Although donanemab is described as a plaque-specific antibody through its recognition of the N3pE epitope enriched in amyloid plaques, it may also bind other N3pE-containing Aβ species, including soluble, insoluble, and intracellular forms [130,131]. By targeting this “seed” form of Aβ, donanemab rapidly clears plaques—a head-to-head trial showed donanemab cleared amyloid faster than aducanumab in early AD patients [20]. Two early-phase clinical trials evaluated donanemab in patients with mild-to-moderate AD. The Phase 1a study (NCT01837641) enrolled 100 participants, while the Phase 1b study (NCT02624778) included 61 participants. Across both trials, donanemab was administered as single and multiple doses, and the results demonstrated that the antibody was generally well tolerated, with no unexpected safety concerns observed [56]. However, ARIA occurred in approximately one-quarter of participants treated with donanemab. A single administration produced measurable reductions in amyloid burden on PET only at doses above 10 mg/kg, whereas repeated dosing at 10 mg/kg and 20 mg/kg achieved sustained decreases in Aβ deposition [132]. Donanemab, a monoclonal antibody targeting the N-terminal pyroglutamate Aβ epitope, was first evaluated in the Phase 2 TRAILBLAZER-ALZ trial (NCT03367403), which enrolled 272 patients with early symptomatic or mild AD and confirmed amyloid and tau pathology. Donanemab, administered every four weeks (700 mg for the first three doses, then 1400 mg), met its primary endpoint with a 3.25-point benefit on iADRS versus placebo, and also reduced amyloid burden by 84 centiloids and modestly slowed tau protein accumulation. ARIA-E and ARIA-H were more frequent with donanemab, though no deaths or serious adverse events occurred. Building on these findings, the Phase 3 TRAILBLAZER-ALZ 2 study demonstrated significant slowing of cognitive and functional decline across multiple endpoints, alongside robust amyloid clearance and attenuation of tau pathology. Supported by these results, donanemab (Kisunla) received FDA approval in July 2024 for early symptomatic AD [133,134], following positive results from the Phase 3 TRAILBLAZER-ALZ 2 trial [17].

Gantenerumab, a fully human monoclonal antibody generated with HuCAL^®^ phage display technology, engineered to selectively recognize and bind Aβ fibrils, targets Aβ fibrils by recognizing amino acids 3–11 and 18–27 of Aβ [135]. In a Phase 1 multiple-ascending-dose study (NCT00531804) of 18 patients with mild AD, intravenous gantenerumab (60 or 200 mg every 4 weeks) reduced cortical amyloid plaques by 15.6% and 35.7%, respectively, compared with placebo. However, two patients in the 200 mg group developed ARIA associated with marked amyloid reduction [136]. A subcutaneous formulation of gantenerumab (105 or 225 mg) was tested in the Phase 2 SCarlet RoAD trial with 490 participants, but the study was discontinued early due to lack of cognitive efficacy [137]. Between this termination and the launch of Phase 3 trials, gantenerumab was also evaluated in the DIAN-TU study (NCT04623242; NCT01760005), which enrolled participants with dominantly inherited AD. In this trial, 52 patients per arm received either gantenerumab or solanezumab, and ARIA-E occurred in 19.2% of those treated with gantenerumab [31]. After the early termination of the SCarlet RoAD trial, the Phase 3 Marguerite RoAD study (NCT02051608) enrolled 389 patients with mild AD, but failed futility analysis and was continued as an open-label extension (OLE). In the combined SCarlet and Marguerite RoAD OLEs, high-dose gantenerumab (1200 mg) reduced brain amyloid by an average of 59 centiloids on florbetapir PET. ARIA-E occurred in about one-third of patients, though most cases were asymptomatic [138]. Following the outcomes of earlier trials, gantenerumab advanced into large Phase 3 testing. In the GRADUATE I (NCT03444870, n = 1053) and GRADUATE II (NCT03443973, n = 975) studies of patients with early AD, gantenerumab failed to demonstrate a significant clinical benefit, with CDR-SB differences of only −0.31 and −0.19 compared with the placebo, despite robust reductions in amyloid plaques and improvements in CSF biomarkers, including total tau, p-tau181, and neurogranin [33]. Long-term open-label extensions of the SCarlet and Marguerite RoAD trials confirmed sustained amyloid clearance, with 80% of patients reaching amyloid negativity after three years [139]. Current development efforts include the Skyline trial (NCT05256134) in cognitively unimpaired, amyloid-positive individuals, and next-generation approaches, such as trontinemab (NCT04639050), a new version of ganterumab, which contains a Fab fragment for better penetration to the BBB. Compared with unmodified ganterumab, 50-folds more trontinemab entered the brain and bound to Aβ plaques with a transferrin receptor–mediated “Brainshuttle™” conjugate of gantenerumab that has demonstrated markedly enhanced brain penetration [140].

AD has a gradual onset and long preclinical course, with pathology developing years before symptoms, so substantial neurodegeneration is often present at diagnosis. Recent anti-Aβ mAbs—aducanumab, lecanemab, and donanemab—have shown that plaque clearance can modestly slow early AD, particularly in patients with MCI or mild dementia. These agents require intravenous infusion and carry class-specific risks of amyloid-related imaging abnormalities (ARIA-E and ARIA-H), especially in APOE ε4 carriers and those on anticoagulants, making MRI monitoring necessary. Most approved anti-Aβ monoclonal antibodies preferentially recognize fibrillar plaque structures, whereas soluble Aβ oligomers—considered to be the more synaptotoxic and diffusible species—are less effectively neutralized, which may limit the extent of cognitive benefit achieved in clinical trials [141]. While clinical benefit remains limited and accessibility constrained by cost and logistics, they represent the first disease-modifying therapies to alter the course of early AD, and ongoing research seeks to improve efficacy and safety [142].

### 4.2. Vaccine

Vaccination approaches aim to elicit an endogenous immune response against Aβ, typically by injecting Aβ peptides (or derivatives) coupled with an immunogenic carrier [143]. The goal is to stimulate the patient’s B-cells to produce anti-Aβ antibodies (without inducing harmful T-cell responses). Vaccines could offer a long-lasting, cost-effective prevention or treatment strategy if successful [144].

AN1792 was the first anti-Aβ vaccine tested in humans, consisting of full-length Aβ42 with the QS-21 adjuvant. In the Phase IIa trial (NCT00021723), about 20% of patients developed high antibody titers, which correlated with reduced amyloid deposition, lower CSF tau protein, and modest cognitive benefits, but only among responders. However, treatment caused T-cell–mediated meningoencephalitis in ~6% of participants, leading to early termination [28,145]. Long-term follow-up later showed that antibody responders maintained low but detectable titers and experienced slower functional decline with sustained benefits [146].

These safety concerns drove the development of second-generation vaccines, such as CAD106 [147] and ACC-001 [148], which used short Aβ fragments or mimotopes to stimulate B-cell responses while avoiding harmful T-cell activation [144]. These candidates demonstrated improved safety and immunogenicity, though clinical efficacy remained limited. Third-generation vaccines, including UB-311 [149], further optimized epitope design and delivery systems, showing favorable tolerability and durable antibody responses in early-phase trials (e.g., NCT00965588, NCT03531710) [149]. More recently, nucleic acid–based approaches (DNA and mRNA vaccines) have entered preclinical testing [150].

DNA vaccines, considered third-generation vaccines, offer several advantages over traditional peptide formulations, including intrinsic safety, cost-effectiveness, and the ability to sustain antigen expression without exogenous adjuvants [151]. AV-1959D is a DNA vaccine encoding three copies of Aβ1-11 fused with 12 foreign T-helper epitopes (derived from tetanus toxin, hepatitis B, and influenza viruses) to enhance immune responses, particularly in elderly populations with weaker vaccine responsiveness [108,152]. Preclinical studies showed that AV-1959D elicited strong anti-Aβ antibody production and robust cellular immunity without T-cell infiltration, while reducing amyloid plaque deposition, attenuating glial activation, and preventing behavioral deficits in aged mice [151,153,154]. Importantly, no toxicities or ARIA were observed in cerebral amyloid angiopathy-prone mice [108]. Based on these findings, AV-1959D entered a Phase I clinical trial in December 2022, designed to evaluate safety and tolerability in 48 participants with early AD [155].

Overall, the trajectory of Aβ vaccine development has moved from full-length peptides to safer, epitope-focused constructs, with the greatest promise likely in early or preclinical AD prevention rather than late-stage treatment [156].

### 4.3. Gene Therapy and RNAi

Unlike antibody therapies, which act extracellularly, RNAi can suppress Aβ production at the transcript level, offering a potentially more durable and upstream method of intervention.

Reducing APP expression offers a therapeutic strategy for AD by limiting substrate availability for Aβ generation. This can be achieved through gene-silencing with intrathecal delivery of siRNA targeting APP mRNA [157]. ALN-APP, the first siRNA therapy developed for CNS diseases, couples synthetic siRNA with a lipophilic 2′-O-hexadecyl (C16) conjugate to enhance CNS penetration [158,159]. In preclinical studies, a single intrathecal dose lowered APP mRNA and sAPPα/β by ~75% in non-human primates, with effects lasting 2–3 months and normalizing by nine months; in APP transgenic mice, treatment reduced APP expression, Aβ40 deposition, and inflammation, while improving synaptic and behavioral outcomes [159]. A Phase 1 trial (NCT04639050) is currently evaluating ALN-APP in patients with MCI or early AD [160]. Interim results released in April 2023 showed dose-dependent reductions of >70% in CSF sAPPα/β sustained for at least three months, with only mild-to-moderate adverse events reported. While multiple-dose cohorts are on hold in the US, they continue in Canada [161].

## 5. Future Perspectives

### 5.1. The “Next Milestone” of Biologics in Aβ Therapy

Future directions in Aβ-based therapy will likely rely on biologics, complemented by nucleic acids, delivery innovations, and precision diagnostics. Five actionable avenues can be outlined:

Earlier intervention relies on precision stratification, combining PET, CSF, and plasma Aβ/p-tau biomarkers, enables identification of at-risk individuals, particularly those with MCI or prodromal AD, while APOE genotype and risk profiling can inform drug eligibility and follow-up intensity [162,163].

Future monoclonal antibody regimens may incorporate personalized dosing and ARIA risk management, while nucleic acid therapies (ASOs, siRNAs) are being explored for low-frequency or long-term dosing with safer routes of administration (e.g., intrathecal, cisterna magna, implantable pumps, or novel BBB-penetrant carriers) [164].

The next wave of trials is expected to test combinations across Aβ, tau protein, and neuroinflammation, leveraging biologics (antibodies, vaccines), nucleic acids (ASOs, siRNAs), and small molecules. Small molecules may remain valuable in genotype-specific or pathway “fine-tuning” niches, exemplified by ALZ-801 targeting APOE4/4 carriers [165,166]. And the innovations in BBB-crossing nanocarriers, ligand-targeted systems, and ultrasound–microbubble technology, combined with brain-targeted chemical modifications, are anticipated to enhance the safety and efficacy of both nucleic acids and next-generation biologics [167].

In summary, the Aβ pathway has not reached its terminus, but has entered a new stage of “precision, multi-modality, and early intervention.” Biologics are likely to remain the backbone, nucleic acids and delivery technologies the accelerators, and small molecules strategic adjuncts in targeted populations and combinatorial regimens.

### 5.2. Aβ Pathways for Future Therapies

Beyond Aβ itself, preclinical research is uncovering upstream and downstream pathways that modulate Aβ pathology. Upstream: APP processing and trafficking—e.g., the role of cholesterol, endocytosis, or autophagy in Aβ generation. Modulating these (as with small molecules enhancing autophagic clearance of Aβ could complement direct Aβ-lowering therapies. Downstream: Aβ and inflammation—preclinical studies show Aβ aggregates activate microglia and astrocytes, contributing to neuroinflammation. Therapies that temper this response (like anti-inflammatory drugs or microglial modulators) might work synergistically with Aβ removal. Co-pathologies: Interactions between Aβ and tau protein—Aβ accumulation appears to accelerate tau phosphorylation and tangle formation. In animal models, combining anti-Aβ and anti-tau protein treatments leads to greater benefits than either alone. This has set the stage for combination trials in humans (e.g., sequential Aβ and tau immunotherapy) [66].

### 5.3. Future Directions

As precision medicine approaches advance, it is clear that not all AD patients are the same. Aβ-targeted therapies may work best in amyloid-positive individuals at mild stages; even among them, factors like APOE genotype, vascular health, and co-pathologies vary. For instance, APOE4 carriers tend to accumulate amyloid earlier but also face higher ARIA risks. Future research should stratify patients to identify who derives the greatest net benefit from Aβ removal. It may be that preclinical AD (amyloid-positive, cognitively normal) individuals will benefit more in the long run than those already with symptoms. Ongoing prevention trials (e.g., the A4 trial, DIAN-TU, API) will inform this, and the field may move toward treating high-risk populations (such as those with familial AD mutations or biomarker-positive MCI) even before clinical onset.

Researchers are already working on the next generation of Aβ therapeutics: more potent antibodies, possibly small molecule amyloid disaggregators, and vaccines for prevention. There is also an emphasis on earlier detection and intervention—integrating routine cognitive assessments with blood biomarker screening in primary care could identify at-risk individuals decades before symptoms, at which point an anti-amyloid vaccine or safe chronic therapy might maintain cognitive health. In parallel, public health efforts (lifestyle interventions, controlling vascular risk factors, etc.) combined with these disease-modifying therapies could collectively reduce the incidence of AD. In summary, targeting the Aβ pathway has opened a door that was closed for many years; stepping through it, the field is cautiously optimistic but recognizes that amyloid is just one piece of a very complex puzzle.

The progress in targeting Aβ provides a roadmap for tackling other aspects of AD. Just as the field persisted with amyloid despite setbacks—eventually achieving success—a similar commitment is now seen in anti-tau therapeutics, neuroinflammation modulators, and more. The coming years will determine how Aβ-targeted drugs integrate into clinical practice and whether early intervention can truly change AD’s trajectory. Importantly, the lessons learned (both triumphs and pitfalls) in the Aβ story will inform all future AD drug development. With improved biomarkers, refined therapeutic approaches, and multi-domain interventions, there is genuine hope that we are moving closer to curbing the global burden of AD. Each advance in the Aβ pathway has not only offered a new treatment option but also deepened our understanding of AD—knowledge that will be invaluable as we strive for a world where Alzheimer’s can be prevented or effectively treated.

## Figures and Tables

**Table 1:** Ongoing clinical trials of small-molecule agents targeting the Aβ pathway in AD.

Agent	Route	Mechanism of Action	Clinical Status (2025)	Reported Efficiency	ClinicalTrials.gov ID	Ref.
ALX-001	Oral	Blocks Aβ–mGluR5 interaction, prevents synapse loss	Phase 1b/2 ongoing	Improved synaptic biomarkers, early safety data	NCT05804383	[85]
RD2 or PRI-002	Oral	Stabilizes Aβ42 monomers, prevents oligomerization	Phase 1/2 ongoing	Preclinical efficacy; early human safety	NCT04711486	[83,84]
Vallitramiprosate (ALZ-801)	Oral	Homotaurine prodrug, inhibits Aβ42 oligomerization	Phase 3 (APOE4/4 AD, ongoing)	Phase 2 showed biomarker benefit; Phase 3 readout 2025	NCT04770220	[81,82]
MIB-626 (NAD^+^ booster)	Oral	Sirtuin-nicotinamide adenine dinucleotide stimulator to enhance alpha-secretase	Phase 2 ongoing	Early biomarker effects	NCT05040321	[86,90]
RQC (Resveratrol Quinone Conjugate)	Oral	Antioxidant + anti-amyloid activity	Phase 1 ongoing	Preclinical efficacy	NCT06470061	[87,88]
RO7269162 (Roche)	Oral	Modulator of amyloid/tau pathways (MOA undisclosed)	Phase 1 ongoing	None yet	NCT06076723	[89]

**Table 2:** Clinical development of biologic drugs targeting Aβ in AD.

Program (Canonical)	Modality	Prevention_or_Treatment and Disease Stage	Primary Target/Epitope	Trial_IDs (NCT No.)	Trial_Status and Regulatory_Status	Ref.
Phase 3						
Lecanemab	Humanized IgG1 monoclonal antibody (IV)	Treatment of early symptomatic AD (MCI/mild dementia); separate prevention in preclinical amyloid+ individuals	Aβ protofibrils (soluble aggregates)	NCT03887455 (CLARITY AD, P3); NCT04468659 (AHEAD 3-45, P3 prevention)	FDA approved (US, July 2023); EU marketing authorisation (April 2025); OLE/real-world and prevention trials ongoing	[55,92]
Donanemab	Humanized IgG1 monoclonal antibody (monthly IV)	Treatment of early symptomatic AD; separate prevention in preclinical amyloid+ individuals	Pyroglutamate Aβ at position 3 (pGlu3-Aβ) on plaques	NCT04437511 (TRAILBLAZER-ALZ 2, P3); NCT05026866 (TRAILBLAZER-ALZ 3, prevention)	FDA approved (US, July 2024); EU CHMP positive opinion (July 2025), EC decision pending (August 2025)	[93,94]
Remternetug	Humanized IgG1 monoclonal antibody (IV)	Treatment of early symptomatic AD	pGlu3-Aβ (N3pE-Aβ) on plaques	NCT05463731 (TRAILRUNNER-ALZ 1, P2); NCT06653153 (TRAILRUNNER-ALZ 3, P3)	Phase 3 active; no marketing approvals	[57,95]
Gantenerumab	Human IgG1 monoclonal antibody (subcutaneous)	Treatment of early symptomatic AD	Conformational epitope spanning Aβ N-terminus + mid-region in fibrils/plaques	NCT03444870 (GRADUATE I, P3); NCT03443973 (GRADUATE II, P3)	Phase 3 (GRADUATE I and II, 2022): failed primary endpoints. Development discontinued; no approvals	[31,57,96]
Solanezumab	Humanized IgG1 monoclonal antibody (IV)	Treatment trials in mild-to-moderate AD; prevention in preclinical amyloid + older adults (A4)	Aβ mid-domain; monomer-preferring	NCT00905372/73 (EXPEDITION 1/2, P3); NCT01900665 (EXPEDITION 3, P3); NCT02008357 (A4 prevention, P3)	Phase 2/3: autosomal-dominant AD trials ongoing; AD efficacy stopped; no approvals	[30,97]
Trontinemab	Bispecific mAb with transferrin-receptor ‘Brainshuttle’ (IV)	Treatment of early symptomatic AD	Aβ fibrils/plaques + transferrin receptor for blood–brain barrier (BBB) transport	NCT04639050 (P1b/2)	Phase 1b/2 multiple-ascending dose; Phase 2 active (China/Australia); no approvals	
Phase 2						
Sabirnetug ACU193	Humanized monoclonal antibody (IV)	Treatment of early symptomatic AD	Aβ oligomers (AβO) (globular soluble species)	NCT06335173 (INTERCEPT-AD, P2); NCT04931459 (P1 completed)	Phase 2 randomized DBPC ongoing; no approvals; FDA Fast Track (2022)	[98,99]
SHR-1707	Humanized IgG1 monoclonal antibody (IV)	Treatment of early symptomatic AD (MCI/mild AD)	Aβ fibrils + monomers	NCT04973189 (retrospectively registered on 21 July 2021) NCT04745104 (registered on 6 February 2021)	Phase 1 and 1b completed; Phase 2 planned; no approvals; development ongoing.	[100]
ABBV-916	Humanized monoclonal antibody (IV)	Treatment of early AD	N3pE-Aβ (pyroglutamate-Aβ) in plaques	NCT05291234 (Phase 2)	Phase 2 started August 2022 (active–not recruiting; est. completion October 2025); no approvals; development ongoing.	[101,102]
ACI-24.060	Liposomal peptide vaccine (active Aβ immunization)	Treatment of prodromal/early AD; separate Down syndrome cohorts	Misfolded Aβ; induces antibodies to oligomers and pGlu-Aβ	NCT05462106 (ABATE, P1b/2)	Phase 1b/2 ongoing; FDA Fast Track; promising preclinical/early clinical results; not discontinued	[103,104]
Phase 1						
ALN-APP	RNA interference (siRNA) delivered intrathecally	Treatment; early symptomatic AD (dose-finding)	APP mRNA	NCT05231785 (Phase 1)	Phase 1 ongoing (active–recruiting; single and multiple dose); no approvals; development ongoing.	[105,106]
ALIA-1758	Anti-pGlu3-Aβ monoclonal antibody with BBB-shuttle (IV/SC)	Therapeutic; early AD (future patient studies)	Aβ aggregates; TfR-mediated BBB transport	NCT06406348 (Phase 1 healthy volunteers)	Phase 1 completed (HVs, May 2024–April 2025; confirmed July 2025); no approvals; development ongoing under AbbVie.	[107]
AV-1959D	DNA vaccine (plasmid)	Treatment of early AD/MCI (dose-escalation)	Aβ1-11 N-terminal epitope (B-cell)	NCT05642429 (Phase 1)	Phase 1 completed in HVs; Phase 1b ongoing in AD patients. no approvals	[108]
CM383	Humanized monoclonal antibody (IV)	Treatment of MCI due to AD/mild AD	Aβ (reported)	NCT06619613 (Phase 1b)	Phase 1 ongoing (HVs; single ascending dose RCT, May 2024–est. June 2025); no approvals	[109]
ALZ-101	Peptide vaccine (AβO-selective)	Treatment of early AD	Aβ oligomers (oligomer-specific)	NCT05328115 (Phase 1b)	Phase 1b completed (mild AD/MCI); safety and immunogenicity endpoints met; no ARIA-E/meningoencephalitis; no approvals; early clinical stage	[110,111]
ION269	Antisense oligonucleotide (intrathecal)	Treatment; early AD	APP mRNA	NCT06673069 (Phase 1/2a)	Phase 1b ongoing (active–recruiting, started December 2024; est. completion March 2027); no approvals; early clinical development	[112]
PMN310	Humanized monoclonal antibody (IV)	Treatment of early AD	Aβ oligomers (AβO)	NCT06105528 (P1a HV); NCT06750432 (P1b AD)	Phase 1b (PRECISE-AD) ongoing (early AD, started 2025); no approvals; development ongoing	[113]

## Data Availability

No new data were created or analyzed in this study. Data sharing is not applicable to this article.
